# Role of the Plant–Microbiome Partnership in Environmentally Harmonious 21st Century Agriculture

**DOI:** 10.3390/microorganisms13122839

**Published:** 2025-12-13

**Authors:** Shashi B. Sharma, Kiran P. Raverkar, Suhas P. Wani, Davis Joseph Bagyaraj, Annapurna Kannepalli, Diwakar R. W. Kandula, Aram Mikaelyan, Minshad A. Ansari, S. Patricia Stock, Keith G. Davies, Rajan Sharma

**Affiliations:** 1 Harry Butler Institute, Murdoch University, Murdoch, WA 6151, Australia; shashi.sharma@murdoch.edu.au; 2 Department of Soil Science, G. B. Pant University of Agriculture and Technology, District Udham Singh Nagar, Pantnagar 263 145, Uttarakhand, India; kraverkar@gmail.com; 3 ICRISAT Development Centre, International Crops Research Institute for the Semi-Arid Tropics, Patancheru, Hyderabad 502324, Telangana, India; wani.suhas@gmail.com; 4 Centre for Natural Biological Resources & Community Development, 41 RBI Colony, Anand Nagar, Bengaluru 560024, Karnataka, India; djbagyaraj@gmail.com; 5 Division of Microbiology, ICAR-Indian Agricultural Research Institute, New Delhi 110 012, India; annapurna96@yahoo.co.in; 6 Faculty of Agriculture & Life Sciences, Lincoln University, P.O. Box 85084, Lincoln 7647, New Zealand; diwakar.kandula@lincoln.ac.nz; 7 Department of Entomology and Plant Pathology, North Carolina State University, Raleigh, NC 27606, USA; amikael@ncsu.edu; 8 Bionema Group Limited, Room 009, Institute of Life Science 1, Swansea University, Swansea SA2 8PP, UK; m.a.ansari@bionema.com; 9 Department of Horticulture, Oregon State University, ALS Bldg. Rm 4007B, 2750 SW Campus Way, Corvallis, OR 97331, USA; patricia.stock@oregonstate.edu; 10 School of Life and Medical Sciences, University of Hertfordshire, Hatfield AL10 9AB, UK; k.davies@herts.ac.uk; 11 Crop Protection and Seed Health, International Crops Research Institute for the Semi-Arid Tropics, Hyderabad 502324, Telangana, India

**Keywords:** microbiome, sustainable agriculture, crop productivity, food security, climate resilience, planetary health

## Abstract

The 21st century calls for a paradigm shift in agricultural practices to address the pressing issues of regeneration of soil health, climate change, environmental degradation, sustainability under growing population pressures, and food security challenges. This article reviews the potential of the plant–microbiome approach as a key driver for eco-conscious green farming. The focus is on the diverse roles of microbial communities in close association with plants in improving plant health, crop productivity, and soil ecosystem functions, and in enhancing environmental sustainability, with focus on four key areas: (1) Soil health and fertility through microbial partnerships; (2) Ecosystem sustainability through microbial functions; (3) Plant health, productivity and food security through microbial innovations emphasising the potential of microbial applications (biofertilisers, bioprotectants, and biostimulants) in sustainable agriculture; (4) Standardisation and stewardship in microbial agriculture highlighting the need for standardisation and quality control in microbial product development and use, and the concept of microbial stewardship and its importance in long-term agricultural sustainability. By synthesising current knowledge and identifying future challenges, this review underscores the transformative potential of the plant-associated microbiome approach in creating resilient, productive, and environmentally harmonious agricultural systems. We highlight current research gaps and future directions, arguing that embracing microbial solutions is not just an option but a necessity for ensuring food security and environmentally benign sustainability in the face of global challenges.

## 1. Introduction

The 21st century presents unprecedented challenges to global agriculture, demanding a paradigm shift from the conventional practices that defined the green revolution of the 20th century. While innovations of that era dramatically increased food production, they have come at a severe environmental cost and affected the sustainability of food systems. Intensive agriculture, characterized by synthetic inputs, monocultures, and heavy machinery, has degraded soil health, disrupted ecosystems, and contributed significantly to climate change [1,2]. Equally important, these practices have weakened the natural partnerships between plants and their microbiomes, reducing plants’ ability to self-regulate nutrient uptake, resist pests, and adapt to climate stresses. As we face the dual imperatives of feeding a growing global population and mitigating environmental degradation, new approaches are urgently needed. This urgency is underscored by the concept of planetary boundaries, which define the safe operating space for humanity within the Earth system [3,4]. These include the following: (1) climate change; (2) rate of biodiversity loss (terrestrial and marine); (3) interference with the nitrogen and phosphorus cycles; (4) stratospheric ozone depletion; (5) ocean acidification; (6) global freshwater use; (7) change in land use; (8) chemical pollution; and (9) atmospheric aerosol loading [3]. Alarmingly, most of these boundaries have already been transgressed, with agriculture being a primary driver of this overshoot. The implications are clear: if we continue on our current path, we risk triggering catastrophic and irreversible environmental changes.

Amidst these challenges, the plant–microbiome partnership emerges as a promising foundation for sustainable agricultural transformation by linking plant physiology and development directly to microbially mediated processes in the rhizosphere. The vast diversity of microorganisms in soil bacteria—fungi, archaea, and more—play a crucial role in nutrient cycling, pest and disease control, and supporting plant growth and resilience [5]. Plants actively shape their rhizosphere communities through root exudates, influencing microbial composition to optimize nutrient availability and defence signalling. With an estimated one trillion microbial species on Earth, of which 99.9% remain undiscovered, the potential for innovation is immense [6]. The economic value of microbial diversity, while difficult to quantify precisely, is likely to reach into trillions of US dollars annually [7]. However, its true worth transcends monetary metrics, encompassing agroecosystem resilience, biodiversity conservation, and global food security. By harnessing the power of the plant and soil microbiome, we can envision agricultural systems that are not only productive but also regenerative and resilient.

The microbiome approach offers solutions to multiple interconnected challenges:

1. Soil Health: Restoring and maintaining soil fertility, minimising reliance on synthetic fertilisers while maintaining productivity.
2. Climate Change Mitigation: Enhancing carbon sequestration in soils and reducing greenhouse gas (GHG) emissions from agriculture.
3. Water Management: Improving water use efficiency and quality through microbial interventions.
4. Pest and Disease Control: Developing microbial alternatives and adjuncts to chemical pesticides.
5. Nutrient Recycling: Facilitating the efficient use and recycling of nutrients in agricultural systems.

**Figure 1:**
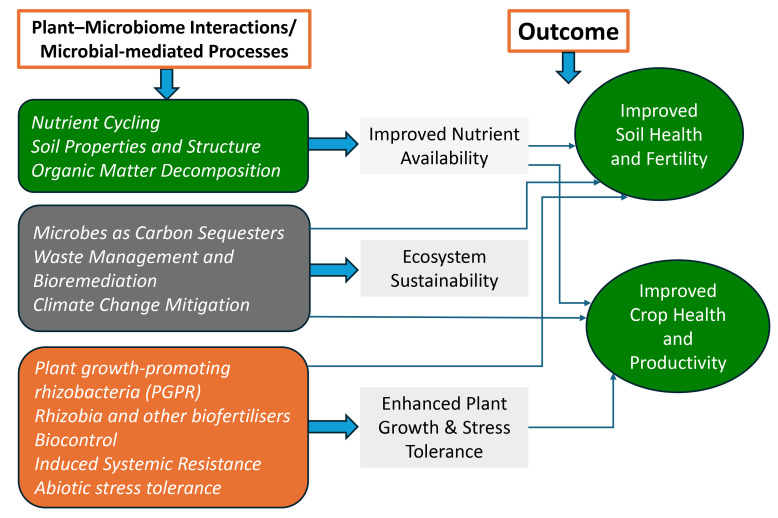
Flow chart of plant–microbiome interactions and outcomes.

This article synthesises the authors’ extensive experience in agriculture, microbiology, and plant and soil health, supplemented by a review of recent literature to elucidate the transformative potential of microbiome-based approaches in agriculture. It highlights the current research gaps and suggests future directions. This article serves as an appeal to scientists, researchers, and policymakers committed to realising the promise of a truly sustainable agricultural future. A flow chart of plant–microbiome interactions and their outcomes is given in Figure 1.

## 2. Soil Health and Fertility Through Microbial Partnerships

Beneath the surface of the earth lies a dynamic and intricate ecosystem, pulsating with life. The soil microbiome, comprising an astounding diversity of bacteria, fungi, archaea, and other microorganisms, forms the foundation of soil and plant health and fertility. Healthy soil microbiomes, shaped and sustained by plant roots, contribute significantly to robust crop growth, higher yields, and long-term agricultural productivity by facilitating nutrient uptake, root development and stress resilience.

### 2.1. Microbial Mediation in Nutrient Cycling and Productivity Enhancement

These microbial communities serve as nature’s own fertiliser factories and soil engineers. By promoting nutrient cycling, improving soil structure, and enhancing water retention, they create an optimal environment for plant growth [8,9]. In monoculture systems, where crop diversity is limited, microbial diversity becomes crucial for ensuring ecosystem resilience [10]. Studies have shown that practices like intercropping, which foster both microbial and functional diversity, can enhance plant growth by up to 15–20% and promote the proliferation of beneficial microbes, such as actinobacteria [10,11]. The nutrient cycle is a fundamental element in agriculture, profoundly influenced by microbial activities. Key players in this process include the following:

1. Nitrogen-fixing bacteria: Convert atmospheric nitrogen into plant-available forms.
2. Nitrifying and denitrifying bacteria: Regulate nitrogen transformation and recycling.
3. Phosphate and potassium solubilising microbes: Convert insoluble nutrients into soluble forms.

For instance, certain *Pseudomonas* strains have demonstrated the ability to increase grain yield by up to 30%, protein content by 15%, and zinc concentration by 25% compared to untreated controls [12]. Arbuscular mycorrhizal fungi (AMF) form symbiotic relationships with plant roots and significantly enhance nutrient uptake. These fungi can extend the effective root surface area by up to 100 times [13,14,15,16], improve phosphorus uptake by 80% in some crops [17,18], and enhance water uptake, particularly crucial during drought conditions [19,20,21,22]. The synergy between AMF and nitrogen-fixing bacteria can increase nitrogen fixation rates by up to 50% in legumes [23,24,25]. Application of a microbial consortium containing *Bradyrhizobium liaoningense* and *Ambispora leptoticha* in soybean cultivation resulted in 19% increase in pod numbers, 34% surge in pod weight per plant, 17% increase in seed count, 32% increase in seed weight per plant, and improved water use efficiency (one of the important drought adaptive traits) and nutrient accumulation over un-inoculated plants under drought stress condition [26]. Recent meta-analytic evidence indicates mean whole-plant nitrogen and phosphorus uptake increases of 67% and ~100%, respectively, with AMF inoculation, albeit context-dependent [27].

### 2.2. Microbial Influence on Soil Properties and Structure

Microbes exert a substantial influence on soil physical and chemical properties, with impacts ranging from the microscopic to the landscape level.

#### 2.2.1. Soil Aggregation and Structure

Soil microbes play a pivotal role in enhancing soil aggregation, which is crucial for maintaining soil structure, improving water infiltration, and fostering root development [28,29,30]. Stronger soil aggregates create better pore networks for roots to explore, enhancing plants’ ability to access both moisture and nutrients. For example, *Pseudomonas* spp. and *Serratia marcescens* produce extracellular polymeric substances, which can increase soil aggregate stability by up to 40% [31]. AMF create hyphal networks that can bind up to 5% of soil carbon, significantly contributing to soil structure [32]. The synthesis of glomalin protein by AMF further enhances micro aggregate stability and maintains soil quality [33].

#### 2.2.2. Organic Matter Decomposition

Microbes actively participate in the decomposition of organic matter, enhancing soil fertility and promoting carbon sequestration. Specific strains of *Streptomyces* have been shown to increase soil organic carbon (SOC) by up to 8%, enhance total nitrogen content by 21%, and improve available phosphorus by 45% [34,35,36].

### 2.3. Research Gaps and Future Directions

**Figure 2:**
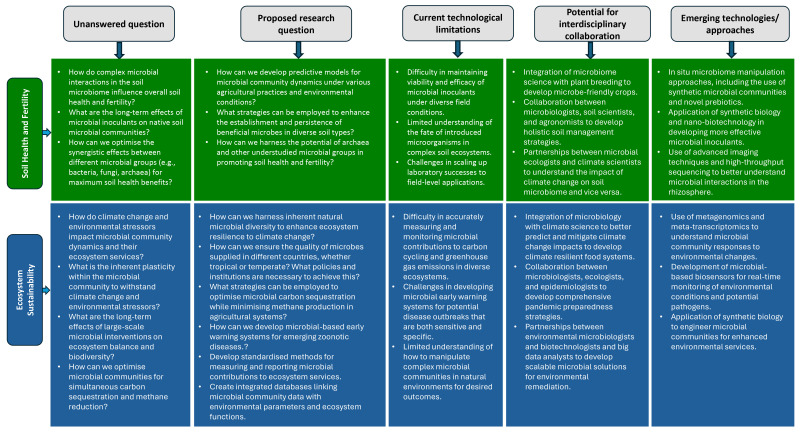
Research gaps and proposed future directions for harnessing the potential of the microbiome for improving soil health and fertility and ecosystem sustainability.

Despite the current body of knowledge, there are key unanswered questions about how plant genotypes, phenology, and root exudation patterns influence microbiome assembly and function in nutrient cycling (Figure 2). By addressing these research gaps and pursuing the proposed directions, which will enhance our understanding of the multitrophic interactions and their synergistic possibilities, we can significantly advance our understanding and application of microbial approaches to improving soil health and fertility. Future research should integrate plant breeding and microbiome management strategies to co-optimize plant traits and microbial functions for soil health and fertility.

## 3. Ecosystem Sustainability Through Microbial Functions

Microbes play a crucial role in maintaining ecological stability, extending their influence beyond agricultural productivity and contributing to the circular economy. In plant-based agricultural systems, these microbial roles are closely tied to supporting plant health, growth, and stress resilience, making them essential allies for sustainable crop production. Understanding their diverse contributions is essential for integrating agricultural activities for ecosystem health.

### 3.1. Microbes as Carbon Sequesters

Microbes significantly influence global carbon dynamics, acting as key players in sequestering atmospheric carbon dioxide. Soil and rhizosphere microbes facilitate carbon cycling through organic matter decomposition, directly and indirectly supporting plant photosynthesis and growth, which in turn sequester carbon globally. Plants, through root exudation, supply carbon-rich compounds that feed these microbial communities, creating a feedback loop between plant productivity and microbial storage.

Management practices like landform modification, cropping systems, and nutrient management can enhance microbial activity and carbon sequestration [37,38]. Microbial inoculation in crop residue-amended soils can increase organic carbon levels by up to 0.83 percentage points. Specific bacterial strains like *Pseudomonas fluorescens*, *Bacillus pumilus*, and *Bacillus pasteurii* contribute to carbon sequestration through various metabolic pathways and carbonate induction [39,40]. Photosynthetically active cyanobacteria are effective in carbon capture, especially under optimal conditions [41,42,43]. In semi-arid cold desert soils, microbial interventions involving *Azotobacter*, *Bacillus subtilis*, and cyanobacteria like *Nostoc* and *Oscillatoria* have significantly increased soil carbon sequestration rates [44,45]. Certain melanised endophytic fungi can enhance carbon content by up to 17% (relative increase) in carbon-rich soils over specific periods [46,47].

### 3.2. Microbes as Soil Erosion Protectors

Microbes play an indispensable role in protecting against soil erosion by promoting stable soil aggregates and improving soil structure. For plants, this means stronger anchorage, improved root exploration, and reduced susceptibility to drought stress.

Microbial secretion of polysaccharides binds soil particles, strengthening the soil matrix [48,49,50]. Biopolymers produced by microbes like *Leuconostoc mesenteroides* and *Sphingomonas paucimobilis* effectively reduce erosion in sandy soils [51,52]. Microbe-induced carbonate precipitation (MICP) can reduce wind erosion rates by 96.5% to 99.5% in sandy desert terrains. Microbial presence increases large and medium soil aggregates by 27.5%, decreasing soil loss and runoff by an average of 93.7% and 68.8%, respectively. *Cyanobacteria*-dominated biocrusts demonstrate remarkable resistance to erosion, reducing sediment concentration by 92–99% and soil loss by 72% [53,54,55,56].

### 3.3. Microbes in Waste Management and Bioremediation

Microbes help turn wastes into useful products and reduce pollution in soils and water used for farming and land care [57,58]. They do this in two main ways: 1. breaking down organic matter into safer, stable forms; and 2. binding or transforming harmful substances so they are less available or easier to remove.

In aerobic composting, mixed microbes heat the pile, break down plant material, and then stabilise it as the pile cools. Good results need enough air and moisture and a balanced mix of fresh (high-nitrogen) and dry (high-carbon) inputs. The finished compost is stable, improves soil structure, and adds nutrients, which helps reduce waste going to landfill or open dumping. Vermicomposting adds earthworms to the process [59,60,61]. Their casts carry a rich microbial community and often increase beneficial microbes in soil, which can support nutrient cycling, plant growth, stress tolerance, and better seedling establishment and root growth, while improving soil structure, provided the feedstock is clean and the process is well managed [59].

Organic pollutants [e.g., hydrocarbons and polycyclic aromatic hydrocarbons (PAHs)]: Some long-lasting organic pollutants, including PAHs, occur in soils, sediments and water near industry and farms. Several microbes can use these compounds as food and break them down [62,63,64,65]. Examples include *Pseudomonas putida*, *Mycobacterium* strain PYR-1 and the fungus *Pleurotus ostreatus*. The white-rot fungus *Coriolus versicolor* can degrade a large share of pyrene, and mixed cultures of *C. versicolor* with *Fomitopsis palustris* can improve this effect [66]. Many basidiomycete fungi are effective because they produce wood-rotting enzymes such as lignin peroxidase and manganese peroxidase that attack complex PAH rings and lower toxicity. Success in the field depends on good contact between microbes and the pollutant and steady conditions (air, moisture and nutrients). Monitoring should check not just concentration drops but also reduced toxicity.

### 3.4. Microbes in Water Management and Wastewater Treatment

As global water demands escalate, the re-evaluation of wastewater emerges as a sustainable and viable resource, pivoting from mere ‘treatment and disposal’ to strategies centered on ‘reuse, recycle, and resource recovery’. Integral to this paradigm shift are plants and, significantly, microbes, especially in the context of constructed wetlands. Constructed wetlands epitomise cost-effective, sustainable solutions for wastewater treatment, especially in resource-constrained rural settings. They not only clean water but also create plant–microbe systems where wetland vegetation and associated microbes jointly remove contaminants. These systems leverage the power of microbes to treat wastewater effectively, extract valuable resources including energy, nutrients, and organic matter, and impact food security, energy sustainability, and climate change mitigation positively [67,68,69]. The Food and Agriculture Organisation (FAO) highlights the immense potential of wastewater in agriculture, as global annual municipal wastewater generation could potentially irrigate 40 million hectares. This area equates to over a quarter of India’s agricultural land. Such reclaimed water reservoirs not only provide viable irrigation solutions but also bolster ecosystem services. For agriculture, this includes providing irrigation-quality water that supports crop production without introducing harmful residues. Through microbial interventions, these systems conserve freshwater resources, rejuvenate aquatic ecosystems, and replenish aquifers. This approach encapsulates a holistic strategy for sustainability [70,71].

### 3.5. Microbes in Climate Change Mitigation

As the spectre of climate change looms large, understanding and leveraging the intricate role of microbes within sustainable agriculture is paramount. Microbes emerge as a potential resource, spearheading the concept of carbon farming - a holistic approach targeting the sequestration of atmospheric carbon dioxide deep within the soil. Certain soil microbes possess a remarkable capacity to convert organic matter into stable carbon compounds, enhancing soil fertility and serving as potent allies in combating climate change. Agricultural methods that promote the flourishing of microbial ecosystems, such as cover cropping, intercropping, and reduced tillage, are indispensable strategies to maximise the effectiveness of carbon farming [38,72]. Cyanobacteria, symbiotically associated with plants, enhance photosynthetic activity and facilitate carbon fixation [73,74,75,76]. Mycorrhizal fungi worldwide sequester an impressive 12 gigatons of carbon dioxide annually from plants, which accounts for 36% of the total current annual carbon dioxide emissions generated by fossil fuels [77]. Rice cultivation, while essential for global food security, is a significant contributor to methane emissions. However, methanotrophic bacteria offer a natural solution. These specialised bacteria oxidise methane, converting it into less harmful forms before it escapes into the atmosphere (Table 1). Integration of methanotrophic bacteria into rice cultivation practices represents an excellent opportunity for sustainable rice farming. Root-associated microbes offer plants an array of benefits that are indispensable for their survival and productivity in challenging environments. These microbial companions enhance the plant’s ability to withstand drought, salinity, and heat stresses, thereby safeguarding agricultural yields [26,78]. Plant growth-promoting rhizobacteria (PGPR) utilise a range of strategies to prepare plants for environmental challenges, including hormonal modulation, gene regulation, osmotic adjustment, and improved nutrient uptake [79].

### 3.6. Microbes in Agricultural Pandemic Preparedness

In the face of potential pandemics, the importance of microbes extends beyond traditional views of infectious diseases. Microbes serve as early-warning systems for animal and plant diseases, revealing changes in their environment and alerting us to potential disease outbreaks [88,89]. This creates a complex connection between agricultural sustainability and pandemic preparedness. Microbes provide ecosystem services to farmers and extension services that benefit long-term ecosystem health [90,91]. Their subtle reactions and changes can indicate early signs of environmental disruptions. The biodiversity and resilience of microbial communities function as bioindicators, providing valuable information about ecological health. Fluctuations in microbial patterns and compositions offer crucial insights into emerging ecological imbalances and potential origins of diseases [92]. Microbial metagenomics has emerged as a potent tool for proactive detection and surveillance of emerging infectious diseases. Metagenomic analyses allow for early detection of potential pathogens by exploring the genetic makeup of microbes in diverse environmental spaces [93,94]. This technology enables proactive microbial surveillance, laying the foundation for resilient early warning infrastructures. By integrating microbial surveillance into overarching disease monitoring paradigms, we enhance our agility in implementing containment and mitigation measures [95,96,97]. It is, however, crucial that microbiome research adopts a planetary health approach, recognising the interconnectedness of human, animal, plant, and environmental health. Studying microbiomes across different species and ecosystems can identify shared microbial reservoirs, transmission pathways, and risk factors for disease transmission. This approach enables collaborative efforts to prevent and control emerging infectious threats at the human–animal–plant–environment interface.

### 3.7. Research Gaps and Future Directions

Despite significant advancements and the vast potential of leveraging microbes to enhance environmental health and sustainability, several critical research questions and gaps remain, particularly in understanding how plant traits (root exudation patterns, phenology, stress physiology) interact with microbial community dynamics to deliver ecosystem services such as carbon sequestration, nutrient cycling, and disease suppression. Plant phenology and stress physiology influence root exudation and habitat filtering, which shape microbial community composition and function; in turn, these community functions contribute to ecosystem services via defined, measurable pathways. Addressing these key areas is crucial for the continued development and application of microbiome-based solutions (Figure 2).

## 4. Plant Health, Productivity and Food Security Through Microbial Innovations

The role of microbes in ensuring food security is multifaceted and crucial. Microbes contribute significantly to enhancing plant health and crop productivity, which are intricately linked to the dynamic interplay between plant traits (e.g., root architecture, exudation patterns), beneficial microbes, environmental factors, soil fertility, and water availability. This plant–microbe interplay directly shapes crop resilience, yield stability, and nutritional quality. Understanding the complexities of these interactions is paramount for establishing a robust scientific foundation to sustainably enhance crop productivity [98].

### 4.1. Biofertilisers, Biostimulants and Bioprotectants: The 3Bs

The microbiome’s integral role in ensuring food security is highlighted by its diverse functions as biostimulants, biofertilisers, and bioprotectants—let us collectively call them ‘microbial 3Bs’. Each of these categories plays a crucial part in sustainable agriculture, contributing to crop health, productivity, and resilience. It is important to note that the definitions of these terms are still evolving as the understanding of microbial contributions to agriculture deepens. Microbial biostimulants produce compounds like phytohormones, amino acids, and enzymes that stimulate plant growth, root development, and nutrient uptake. Mycorrhizal fungi, for instance, form symbiotic relationships with plant roots, enhancing nutrient uptake and improving overall plant health and stress tolerance. Microbial biofertilisers include microbes such as nitrogen-fixing bacteria like *Rhizobium* and *Azotobacter*, and phosphate-solubilising bacteria such as *Pseudomonas* and *Bacillus*, which contribute significantly to nutrient cycling and availability in the soil. These microbes convert atmospheric nitrogen into usable forms for plants or solubilise insoluble phosphates, reducing the need for synthetic fertilisers and minimising nutrient runoff and soil degradation. Microbial bioprotectants offer eco-friendly alternatives to chemical pesticides by directly antagonising pests and pathogens or inducing systemic resistance in plants. For example, *Bacillus thuringiensis* [Bt] produces insecticidal proteins toxic to certain pests, while certain strains of *Trichoderma* fungi act as biocontrol agents, suppressing soil-borne pathogens through competition and the production of antifungal compounds. Their effectiveness often depends on plant genotype–microbe compatibility, making plant breeding for symbiosis a valuable complementary strategy.

#### 4.1.1. Plant Growth-Promoting Rhizobacteria (PGPR)

PGPR, encompassing various representatives such as *Azotobacter*, *Bacillus*, *Pseudomonas*, and *Rhizobium*, play a significant role in enhancing plant health and growth. Their functions span from atmospheric nitrogen fixation and synthesis of vital phytohormones to mineral solubilisation and inhibition of pathogens. Integrating them into agricultural practices presents a promising solution for increasing crop productivity [99,100]. Research across various crops, including cereals, legumes, fruits, vegetables, herbs, and ornamentals, consistently demonstrates the benefits of these PGPR strains. However, large-scale production and commercialising these bioresources remains a challenge, particularly in many developing nations, due to infrastructural and regulatory constraints [101]. The emerging terminology ‘plant probiotics’ simplifies the concept, emphasising their symbiotic relationship with plants [102]. Biological nitrogen fixation research, particularly by institutions like the International Crops Research Institute for the Semi-Arid Tropics (ICRISAT) and the Indian Agriculture Research Institute (IARI), has highlighted the profound impacts of PGPR in enhancing soil and plant health and significantly increasing crop yields [103,104,105]. In practice, PGPR perform best when embedded in good agronomy—seed quality, balanced fertilisation and irrigation timing—so that microbial benefits add to, rather than substitute for, the basics of crop management.

#### 4.1.2. Rhizobia and Other Biofertilisers

Biofertiliser formulations, enriched with beneficial microorganisms such as *Rhizobium* and *Azotobacter*, offer a promising eco-friendly alternative. Their key strength lies in their capacity to fix nitrogen, solubilise phosphates and potassium, and promote vigorous plant growth [106]. These microbes play a versatile role in enhancing root architecture, seed germination, and plant biomass, while also bolstering plant resistance against various diseases. Their symbiotic relationships with plants significantly boost crop yields while minimising environmental harm [107,108,109]. Species like *Burkholderia cepacia* and *Pantoea rodasii* stand out for their versatile plant growth-promoting capabilities through the solubilisation of essential nutrients such as potassium, phosphorus, nitrogen, and zinc. Moreover, these bacteria contribute to plant health through the production of siderophores, the synthesis of plant growth hormones like Indole-3-acetic acid [IAA], and ammonia production. Research highlights their effectiveness in enhancing the growth and yield of vital crops like paddy [110] and black gram [111], offering a tangible pathway towards sustainable agricultural practices balancing productivity with ecological responsibility (Table 2). The true potential of biofertilisers is further magnified when deployed as a synergistic microbial consortium. (Table 2)

**Table 2:** Beneficial microbes and their contribution to various crop improvement traits.

Microbe	Crop	Role	Reference
*Pseudomonas protegens*	Wheat (*Triticum aestivum*)	Increased the IAA/ABA ratio, enhanced grain yield	[112]
*Bacillus cereus* ZnSB13	Chickpea (*Cicer arietinum* L.)	Increment in fresh and dry nodule weights, shoot and root dry weight, effective grain yield	[113]
*Pseudomonas* spp., VBZ4	Tomato (*Solanum lycopersicum* L.)	Taller plants, broader stems, higher fresh and dry shoot and root weights, and a greater number of fruits per plant	[114]
*Rhizobium tropici* + *Bacillus subtilis*	Common bean (*Phaseolus vulgaris* L.)	Improvement in shoot dry matter and grain yield	[115]
*Gluconacetobacter diazotrophicus*	Rice (*Oryza sativa*)	Promoting various root growth and developmental mechanisms	[116]
*Pseudomonas fluorescens*	Foxtail millet (*Setaria italica*)	Increase in germination rate, improved soil adherence to root tissue, dry mass	[117]
*Pseudomonas jessenni* MP1 and *Pseudomonas palleroniana* N26	Kidney bean (*Phaseolus vulgaris* L.)	Improved grain yield, nutritional status, and crop growth	[12]
*Streptomyces griseus* CAI-24	Chilli and tomato	Enhanced fruit yield	[118]
*Rhizobiapusense* IC-59 and *Paraburkholderia kururiensis* IC-76a	Chickpea (*Cicer arietinum* L.)	Enhanced nodule number and nodule weight and IAA production	[119]
*Streptomyces avermitilis* CAI-85 and *Streptomyces albus* CAI-93	Rice (*Oryza sativa*)	Root and shoot development and crop productivity	[120]
*Streptomyces* spp., CAI-26 and *Streptomyces griseus* MMA-32	Pigeonpea (*Cajanus cajan*)	Enhanced stover and grain yield	[121]
*Streptomyces griseus* CAI-24 and *Streptomyces griseus* MMA-32	Pearl millet (*Pennisetum glaucum*)	Enhanced the stover and grain yield and improved nutrient content (Iron and Zinc)	[122]
*Streptomyces griseus* CAI-68 and *Streptomyces griseus* MMA-32	Chickpea (*Cicer arietinum* L.)	Increased seed mineral content	[123]
*Streptomyces africanus* KAI-32	(Sorghum) *Sorghum bicolor*	Significantly enhanced stover yield	[124]
*Trichoderma* spp.	Sugarcane, rice, vegetables	Biocontrol; enhanced nutrient uptake	[125]
AMF	Cotton	Enhanced phosphorus acquisition, growth, seed cotton yield and fiber quality	[126]
Rhizobia	Legumes	Biological nitrogen fixation, phytohormone production and stress resilience	[127]

In legume-centric cropping systems, nitrogen-fixing rhizobia, present a sustainable alternative to conventional chemical fertilisers. Complementing them, biostimulants increase plant growth and resilience by leveraging microbial metabolites, thereby reducing dependency on synthetic fertilisers and augmenting soil health [128]. Field studies with AM fungi have shown excellent effects in boosting the growth and yield of various crops, including tomatoes, chillies, soybeans, cowpeas, and potatoes, by an impressive margin of 14–20% with a 50% reduction in phosphate fertiliser usage. The synergistic effect with other beneficial microbes becomes even more pronounced, enhancing both plant growth and yield. This synergy not only reduces the need for fertiliser applications but also improves soil health [25]. The symbiotic integration of biofertilisers and biostimulants provides a holistic strategy to boost crop productivity rooted in strengthening the plant–microbe nexus rather than relying solely on external chemical inputs while simultaneously mitigating the environmental impacts linked with current farming practices. This approach paves the way for a more sustainable and resilient agricultural landscape [128,129]. The intricate partnership between microbes and crops holds the potential for remarkable improvements in both yield and sustainability. Taking a closer look at specific examples, the use of *Rhizobium* has played a crucial role in enriching the soil with nitrogen, leading to increased crop yields, especially benefiting subsequent cereal crops [130].

#### 4.1.3. Arbuscular Mycorrhizal Fungi (AMF)

AMF extend the effective absorptive surface of roots via extra-radical hyphae, improving phosphorus capture and water relations. Benefits are strongest in low-to-moderate phosphorus availability, with diminishing returns at high soluble phosphorus. Beyond nutrient effects, AMF can alter root system architecture and phenology, often advancing reproductive development. Successful on-farm use turns on compatible host genotypes, inoculum quality, and fertiliser placement that does not suppress colonisation. Further accentuating this narrative, a case study elucidates the transformative potential of microbial consortia in chilli [*Capsicum annuum*] cultivation. Through synergistic interactions between *Bacillus sonorensis* and AM fungus *Funneliformis mosseae* (formerly* Glomus mosseae)*, not only were growth and yield amplified, but soil health also improved. Astonishingly, this microbial intervention facilitated a 50% reduction in chemical fertiliser usage without compromising chilli’s quality or yield [131].

#### 4.1.4. Microbes as Bioprotectants

Leveraging the potential of beneficial microbes, particularly fungi such as *Trichoderma* and *Clonostachys* (formerly *Gliocladium*), offers a sustainable approach to disease management in plants and promotes eco-friendly agricultural practices [132,133]. Moreover, PGPR indirectly boost plant health by producing allelochemicals and bioactive molecules such as siderophores, lytic enzymes, and antibiotics. These substances effectively combat plant pathogens [134,135]. The allelochemicals, including antibiotics, inhibit the growth of harmful bacteria and fungi, ensuring plant protection even at minimal doses. Enzymes such as chitinases, cellulases, and proteases from PGPR break down cell walls of pathogenic fungi, halting their proliferation [135]. PGPR also initiates systemic resistance in plants against pathogens through the elicitation of physical and chemical changes related to plant defence. Several substances, including jasmonate, flagellar proteins, ethylene, pyoverdine, chitin, N-alkylated benzylamines, β-glucans, cyclic lipopeptide surfactants, N-acyl-homoserine lactones and salicylic acid, are involved in the signalling pathways by which PGPRs induce systemic resistance. The identification of elicitors, such as volatile organic compounds (VOCs) and microbe-associated molecular patterns (MAMPs), can initiate ISR, which can subsequently cascade into a broad-spectrum systemic response across the plant. This induced systemic resistance (ISR) equips plants to more effectively combat the harmful effects of plant pathogens. While ISR’s effects on improved plant resistance to microbial diseases are well established, there is growing interest in ISR’s potential role in defence against insect pests. ISR is mostly triggered when specific conditions are met and is dependent on environmental factors that might change the outcome of plant–microbe interactions. However, one of the main obstacles to the development of ISR-inducing microorganisms as a future agricultural technology is the unpredictable nature of ISR events [136]. Specific strains of actinomycetes and bacteria that exhibit antagonistic properties against common pathogens have been identified, highlighting their potential in plant disease management. By sustaining healthy plant–microbe interactions, these approaches contribute to integrated pest and disease management systems that are less disruptive to agroecosystem balance.

Biologically active secondary metabolites (SMs) present another avenue for innovative control methods. These SMs, produced by microorganisms, exhibit antagonistic effects against plant parasites, pests, and pathogens, playing roles in defence, competition, regulation, and communication [137,138,139,140]. As interest in eco-friendly alternatives to synthetic pesticides grows, microbial bioprotectants emerge as a viable solution [141]. For instance, endophytic fungi exhibit antimicrobial properties against various plant pathogens like *Rhizoctonia solani* and *Fusarium oxysporum* by their ability to produce an array of enzymes for maintaining plant health and managing diseases effectively [142]. Microbial bioprotectants leverage the potential of beneficial microbes, including entomopathogenic fungi and bacteria, to safeguard crops from pests, offering a solution devoid of the harmful effects often linked with chemical pesticides [143,144,145,146]. Numerous examples exist, including *Bacillus thuringiensis* [Bt] toxins that target specific insect pests and *Trichoderma* species proficient in combating fungal diseases [143,147]. Further, PGPR like *Bacillus*, *Pseudomonas*, and *Streptomyces* serve as essential plant defenders against pests [Table 3]. Their versatile mechanisms include the release of insecticidal toxins and metabolites and contribute to enhanced plant resilience [148]. The intricacies of these mechanisms, including protein toxins, lytic enzymes, and secondary metabolites, highlight their potential in sustainable agriculture. For instance, certain compounds derived from *Streptomyces* spp. have demonstrated significant pest mortality rates in crops like chickpea and pigeonpea [149,150]. However, despite their efficacy, scaling up the production of these biocontrol agents remains a challenge. Soil-borne pathogens, particularly nematodes, pose significant threats to crop health. The natural antagonists, including nematode-trapping fungi and bacterial parasites like *Pasteuria penetrans*, have gained attention for their potential role in nematode management. Moreover, advancements in biotechnology have led to the development of microbial biocontrol agents sourced from organisms like *B. thuringiensis* and *Streptomyces avermitilis*. These agents, particularly secondary metabolites from *S. avermitilis*, such as avermectin, have transitioned from mere scientific curiosities to practical solutions. Avermectin, for instance, has carved a niche for itself in the realm of biological control by effectively managing plant-parasitic nematodes. While challenges related to solubility and soil particle absorption persist [151], innovative applications like seed coatings present promising avenues for enhancing efficacy, as observed in cotton cultivation [152]. Historically, synthetic pesticides have been the go-to solution for pest control, but their detrimental environmental impacts are undeniable. These chemicals disrupt soil ecosystems, harm beneficial organisms, and compromise overall soil health [153].

**Table 3:** Some examples of microbial bioprotectants and their mechanisms of action for the control of plant pathogens and insect pests.

Microbe	Test Plant/Disease/Pest	Target Pathogen/Pest	Mechanisms	Reference
*Bacillus amyloliquefaciens*	Rhizome rot disease of turmeric (*Curcuma longa*)	*Rhizoctonia solani*	Antifungal lipopeptides Synthesis	[154]
*Pseudomonas* sp. CMR12a	Root rot of cocoyam (*Colocasia esculenta*)	*Pythium myriotylum*	Phenazines and cyclic lipopeptides synthesis	[155]
*Bacillus thuringiensis*	Sclerotiniose in mustard (*Brassica campestris*)	*Sclerotinia sclerotiorum* and *Plutella xylostella*	ISR in plants by simultaneously activating SA, JA, and ET signaling pathway	[156]
*Pseudomonas fluorescens* Q2-87	*Arabidopsis thaliana*	*Botrytis cinerea*	ISR, phenolic compound synthesis	[157]
*Pseudomonas chlororaphis* R47	Late blight of potato (*Solanum tuberosum*)	*Phytophthora infestans*	Production of HCN inhibiting mycelium and zoospore germination inhibition	[158]
*Paenibacillus polymyxa*	Charcoal rot of soybean (*Glycine max*)	*Rhizoctonia bataticola*	Antifungal lipopeptides Synthesis	[159]
*Paenibacillus polymyxa*	Fusarium wilt of cucumber (*Cucumis sativus*)	*Fusarium oxysporum* f. sp. *cucumerinum*	Antimicrobial compounds & hydrolytic enzyme synthesis	[160]
*Serratia marcescens ETR17*	Root rot of tea (*Camellia sinensis*)	*Lasiodiplodia theobromae*	Production of hydrolytic enzymes	[161]
*Streptomyces violaceusniger* AC12AB	Common scab of potato (*Solanum tuberosum)*	*Streptomyces scabies*	Antibiotic production	[162]
*Trichoderma asperellum* FJ035	Fusarium wilt of cucumber (*Cucumis sativus*)	*Fusarium oxysporium*	Antagonism and spatiotemporal competition	[163]
*Trichoderma* *harzianum*	Curvularia leaf spot of maize (*Zea mays*)	*Curvularia lunata*	ISR to fungal disease	[164]
*Beauveria bassiana*	Cotton leafworm in cotton (*Gossypium *sp.)	*Spodoptera litura*	Pupal & adult deformities	[165]
*Metarhizium anisopliae*	Fusarium head blight of wheat (*Triticum aestivum*)	*Fusarium graminearum*	Produces fungistatic secondary metabolites	[166]
Podoviruses	Soft rot of potato (*Solanum tuberosum)*	*Pectobacterium carotovorum*	Cell lysis	[167]
*Streptomyces* spp., AUR-2	Botrytis gray mold (BGM) of chickpea	*Botrytis cinerea*	Anti-fungal activity and host plant resistance	[168]
*Streptomyces africanus* KAI-32 and *Streptomyces griseus* CAI-127	*Fusarium* wilt of chickpea	*Fusarium oxysporum*	Anti-fungal activity and host plant resistance	[169]
*Streptomyces albus* CAI-21	Charcoal rot of sorghum	*Macrophomina phaseolina*	Anti-fungal secondary metabolites synthesis	[170]
*Streptomyces griseus* CAI-155	Legume pod borer in chickpea	*Helicoverpa armigera*	Antifeedant, larvicidal activity	[149]

In contrast, bioprotectants can offer a sustainable alternative. They are eco-friendly, specific in action, and leave minimal residues [171,172]. Endophytic bacteria and fungi, traditionally viewed as mere plant residents, are emerging as key players in plant health. They produce bioactive metabolites, engage in intricate signalling pathways, and actively combat pathogens, enhancing plant resilience [159,173,174]. They employ diverse strategies for disease control. Whether it is *Azospirillum* enhancing rice defence mechanisms or *Rhizobacteria* and rhizosphere fungi producing antibiotics, the arsenal of microbial tools is vast and versatile [175,176]. However, care must be taken to ensure that there are no negative effects of the bioprotectants on humans.

Tackling post-harvest losses is crucial for enhancing food security, and microbes prove to be powerful allies in this effort. Spoilage agents often contribute to substantial post-harvest losses, but the strategic use of beneficial microbes, such as biocontrol agents, offers a sustainable solution. With their ability to combat pathogens, microbes not only prolong the shelf life of food but also diminish the reliance on chemical preservatives. This microbial-focused approach strengthens resilience in our food systems, reducing losses and optimising resource utilisation [5,177,178].

#### 4.1.5. Economic Impact and Market Growth of 3Bs

Reflecting the potential of 3Bs, international projections indicate a burgeoning biological agriculture sector. Specifically, the global biological agriculture market, encompassing bioprotectants, biostimulants, and biofertilisers, witnessed a valuation of USD 12.9 billion in 2022. Forecasts suggest this figure could soar to an estimated USD 24.6 billion by 2027, marking a notable compound annual growth rate of 13.7%. The bioprotectants sector is poised for an impressive growth trajectory, targeting over USD 11 billion by 2027, propelled by a robust growth rate of 15.6%. Concurrently, the biostimulants market anticipates scaling to USD 6.2 billion, while biofertilisers are on track to touch USD 4.5 billion by 2026 [179]. These projections, of which microbes are an integral part, not only accentuate the escalating demand for biological solutions but also indicate agriculture’s evolving landscape, primed for sustainable innovations. This rapid growth reflects increasing recognition that plant-centred agricultural solutions depend on optimising beneficial microbial inputs as much as on improving crop genetics.

### 4.2. Insect Microbiomes as Allies in Agricultural Ecosystems

Insects have evolved complex symbiotic relationships with microbes to manage their dietary and environmental challenges. These relationships can have profound consequences for plants, influencing pollination, pest pressure, and crop health. The relationships between microbes and their hosts come in various forms, serving different purposes depending on where they are and what they do. For instance, some microbes, like *Buchnera* found in aphids, live inside cells and help by providing essential amino acids and other nutrients. This mutualistic relationship was highlighted in research by Feng et al. [180]. On the other hand, there are diverse microbiomes in the environment and gut that perform various functions. For example, in coffee borers, microbes aid in detoxifying caffeine, a process elucidated by Ceja-Navarro et al. [181]. In wood-feeding insects, such as termites, microbes simplify the decomposition of tough materials like lignocellulose. Various studies further emphasise the role of these microbes in breaking down wood components, facilitating the insects’ digestion and survival. [182,183,184]. In contrast to such internal symbioses, insects rely on microbial partners to manage imbalanced diets, detoxifying, simplifying, and supplementing dietary components. Pests like mealybugs and scale insects rely heavily on obligate symbionts for survival. In contrast, certain pests, such as bark beetles and wood wasps, harness external fungal symbionts to decompose and kill living trees [185]. The complex interplay between insect microbiomes and plants has significant ramifications for agricultural productivity. Insect-associated microbes can modulate plant defences, influencing their perception and subsequent anti-herbivore responses [186]. This modulation can either enhance plant defence against insect feeding or, in some pest cases, suppress those defences, increasing crop vulnerability. Such alterations can exacerbate crop damage by enhancing insect herbivory. Moreover, the influence of microbiomes on insect behaviour and fitness indicates potential shifts in pest population dynamics, posing challenges for crop management. Further complicating this dynamic are entomopathogens, which, when present as endophytes in plants, can alter plant defences and quality. Such alterations can reshape insect–plant interactions, potentially compromising plant health.

Harnessing knowledge about insect microbiomes can inform strategies that mitigate pest-induced crop damage while preserving beneficial insect–plant relationships. By deciphering the intricate web of insect–microbe–plant interactions, researchers and farmers can devise more resilient and sustainable practices for enhancing crop production.

### 4.3. Microbes as Drought-Stress Alleviators

Drylands confront escalating challenges of drought, salinity and heat stress, exacerbated by the ramifications of climate change, including heightened aridity and diminished precipitation events that reduce crop productivity, endangering food security and agricultural sustainability. These stresses directly limit plant growth, reproductive success, and yield stability, making microbially mediated tolerance mechanisms increasingly vital. Amidst these challenges, microbes offer a strategic intervention against drought stress, ensuring the endurance and productivity of crops in arid regions through Rhizobacterial-Induced Drought Endurance and Resilience [RIDER]. This mechanism orchestrates a suite of physiological and biological alterations within plants to bolster their resilience against drought-induced adversities. Table 4 outlines PGPR species that confer plant resilience to abiotic stresses, with emphasis on physiological and metabolic adjustments in the plant host. The PGPRs modify phytohormone levels, antioxidant defence, bacterial exopolysaccharides [EPS], and metabolic adjustments encompassing accumulation of several compatible organic solutes like sugars, amino acids and polyamines. They also produce heat-shock proteins [HSPs], dehydrins and volatile organic compounds [VOCs] which play a significant role in the acquisition of drought tolerance and orchestrate metabolic events within plants during periods of stress [76]. By meticulously screening, selecting, and deploying drought-resilient PGPR strains, it becomes feasible to transcend the constraints of drought stress and optimise crop yields.

**Table 4:** Role and function of microbes in abiotic stress alleviation.

Microbe	Crop	Stress Type	Function	Reference
Species of *Gracilibacillus*, *Staphylococcus*, *Virgibacillus*, *Salinicoccus*, *Bacillus*, *Zhihengliuella*, *Brevibacterium*, *Oceanobacillus*, *Exiguobacterium*, *Pseudomonas*, *Arthrobacter*, *Halomonas*	Maize (*Zea mays*)	Salinity	ACC deaminase, IAA and exo-polysaccharide production; biofilm formation and phosphate solubilization	[187]
*Azospirillum* and *Rhizobia*	Soybean (*Glycine max*)	Moderate Drought	Production of phytohormones and improving root architecture	[188]
*Bacillus aryabhattai*	Cowpea (*Vigna unguiculata*)	Drought and Paraquat pesticide residue	Production of phytohormones and improving root architecture	[189]
*Gigaspora margarita*, *Funneliformis mosseae *, *Funneliformis fasciculatus *	Soybean *(Glycine max)*	Water stress	Vesicular-arbuscular mycorrhizal fungi provided low resistance pathway for water movement across the root	[190]
*B. thuringiensis*	French lavender *Lavandula dentata*	Drought	Production of IAA by the bacterium improving nutrition, physiology, and metabolic activities of plant by inducing higher proline and K-content, and decreased oxidative stress-related enzymes	[191]
*Pseudomonas putida* GAP-P45	*Arabidopsis thaliana*	Water stress	Changes in proline metabolic gene expression profile—up-regulation of the expression of genes involved in proline biosynthesis, i.e., ornithine-Δ-aminotransferase (OAT), Δ 1 -pyrroline-5-carboxylate synthetase1 (P5CS1), Δ 1 -pyrroline-5-carboxylate reductase (P5CR), as well as proline catabolism, i.e., proline dehydrogenase1 (PDH1) and Δ 1-pyrroline-5-carboxylate dehydrogenase (P5CDH).	[192]
*Burkholderia cepacia, Promicromonospora* sp. and *Acinetobacter calcoaceticus*	*Cucumis sativus*	Salinity and Drought	Enhanced leaf biomass and chlorophyll	[193]
PGPR consortia (*Pseudomonas composti* SDT3, *Aeromonas aquariorum* SDT13, *Bacillus zhangzhouensis* HPJ40, *Pseudarthrobacter oxydans* SRT15, *B.methylotrophicus* SMT38 and *B. aryabhattai* SMT48	Grapevine (*Vitis vinifera*)	Heat stress	Osmoprotectant promotion, improved antioxidant mechanisms and membrane stability, improved light-harvesting capabilities	[194]
*Microbacterium* sp. (AR-ACC2), *Methylophaga* sp. (AR-ACC3), and *Paenibacillus* sp. (ANR-ACC3)	Rice (*Oryza sativa*)	Flooding stress	Enhanced germination, seedling vigour index, root and shoot length and total chlorophyll contents, reduced ethylene production	[195]
*Bacillus cereus* VBE23	Maize (*Zea mays*)	Drought stress	EPS and ACC deaminase production; phosphate and zinc solubilization	[196]

The potential and mechanisms of these microbial allies in bolstering plants against challenges induced by drought have been well demonstrated [197,198,199,200,201].

### 4.4. Research Gaps and Future Directions

**Figure 3:**
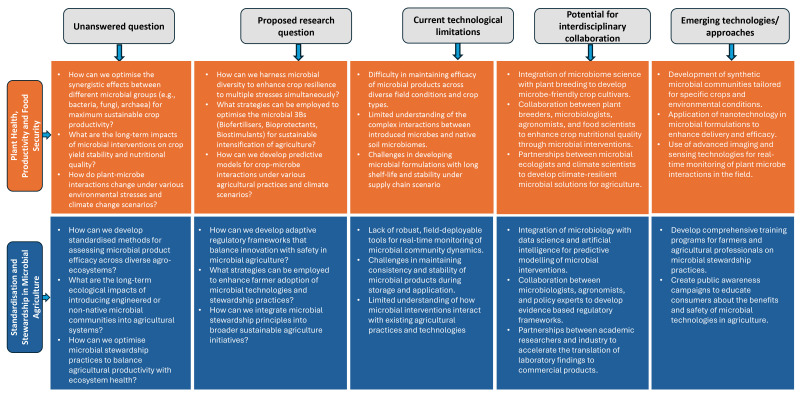
Research gaps and proposed future directions for harnessing the potential of microbiome for improving plant health, productivity and food security, and standardisation and stewardship in microbial agriculture.

Despite significant advancements and the vast potential of leveraging microbes to sustainably enhance crop productivity and food security, several critical research questions and gaps remain, particularly in linking specific plant traits (root exudate chemistry, phenological timing, canopy architecture) with microbial community structure and function under field conditions. Future research should aim to integrate plant breeding, agronomy, and microbial ecology to design crop–microbe systems optimised for yield, resilience, and sustainability. Addressing these key areas is crucial for the continued development and application of microbiome-based solutions for sustainable futures (Figure 3).

## 5. Advancing Standardisation and Stewardship in Microbial Agriculture

### 5.1. Reimagining Standardisation for Microbial Production, Storage, and Utilisation

While some nations have established standard practices and protocols, a significant gap exists in cross-country harmonisation in implementing standardised practices for bioinoculants. This disparity necessitates a paradigm shift in our approach to standardisation. For plant-based agriculture, such standards must explicitly account for the compatibility of microbial products with specific crop species, varieties, and growth stages to maximise plant benefits.

#### 5.1.1. Adaptive Standardisation Frameworks

Current static standards fail to account for the dynamic nature of microbial communities. We propose the development of “living standards”—adaptive frameworks that evolve with scientific advancements and changing environmental conditions. These standards should be flexible enough to accommodate regional variations while maintaining core quality benchmarks.

#### 5.1.2. Standardisation of Complex Microbial Consortia

A critical gap exists in standardising complex microbial consortia versus single-strain products. Research is needed to develop methodologies for characterising and maintaining the stability of multi-strain formulations, considering their synergistic effects and potential shifts in community composition.

#### 5.1.3. Blockchain-Enabled Traceability

To enhance transparency and quality control, we propose integrating blockchain technology into the microbial supply chain. This would enable auditable tracking of production, storage, and application processes, ensuring adherence to standards and facilitating rapid response to quality issues.

In this programme, blockchain is used narrowly as a tamper-evident log of consent, provenance and benefit-sharing for microbial inputs (e.g., inoculant strain identity, lot/batch and supplier; local stewardship agreements) and for recording permissions governing trial datasets. We apply data-minimisation, sovereign hosting of underlying datasets, and open standards for interoperability with farm information systems and extension services. Where smart contracts are applied, they are confined to licensing and revenue-share events and do not trigger agronomic actions. This keeps the chain as an audit trail, not a decision engine, and aligns with national policy and conflict-sensitive deployment.

#### 5.1.4. AI-Driven Quality Assurance

Artificial Intelligence offers untapped potential in quality control. Machine learning algorithms could analyse vast datasets of microbial characteristics, predicting efficacy and identifying potential issues before they arise. This proactive approach could support quality assurance in microbial agriculture.

AI is framed as decision support, not automation. Models are documented (model cards), trained on provenance-aware datasets and calibrated to local context; outputs surface uncertainty ranges and link back to measured inputs (e.g., soil P, moisture, AMF colonisation). Recommendations are reviewed by an agronomist or grower before action, with high-risk cases requiring dual sign-off. All data flows follow consent and data-sovereignty rules; logs enable independent audit. Fail-safe defaults and opt-out paths ensure safe operation if AI services degrade or data permissions change.

#### 5.1.5. Global Microbial Product Database

We advocate for the establishment of a comprehensive, open-access database of microbial products. This resource would facilitate cross-border knowledge exchange, accelerate research, and promote the development of globally applicable standards.

### 5.2. Microbial Stewardship

Microbial stewardship extends beyond responsible use; it encompasses the ethical implications and long-term consequences of manipulating microbial ecosystems.

#### 5.2.1. Microbial Ecosystem Engineering

We propose a shift from isolated microbial applications to “microbial ecosystem engineering.” This holistic approach considers the entire soil microbiome, aiming to create balanced, resilient microbial communities rather than relying on single-strain interventions.

#### 5.2.2. Monitoring Unintended Ecological Consequences

Large-scale microbial applications may have unforeseen impacts on native microbial communities and broader ecosystems. We urgently need novel monitoring strategies, potentially leveraging environmental DNA (eDNA) sequencing and AI-driven predictive modelling to detect and mitigate unintended consequences.

#### 5.2.3. Synthetic Biology and Gene Editing in Microbial Stewardship

The intersection of microbial stewardship with synthetic biology and gene editing presents both opportunities and ethical challenges. Research is needed to explore how these technologies can enhance beneficial microbial traits while establishing safeguards against potential risks.

#### 5.2.4. Global Microbial Diversity Bank

We support the establishment of a global microbial diversity bank to preserve and study microbial strains from various ecosystems. This resource would be invaluable for understanding microbial diversity, identifying novel beneficial strains, and potentially restoring degraded soil ecosystems.

#### 5.2.5. Cross-Kingdom Interaction Studies

A significant knowledge gap exists in understanding how microbial interventions affect plant–insect–microbe interactions. Comprehensive studies on these cross-kingdom interactions are crucial for predicting the full impact of microbial applications in agricultural ecosystems.

### 5.3. Research Gaps and Future Directions

There are several research questions and gaps that need to be addressed. Thus, future research should concentrate on the key areas described in Figure 3.

## 6. Conclusions

The microbiome approach to environmentally harmonious food security offers a pathway to harmonise productivity with environmental stewardship. Framing plants as active participants in shaping and benefiting from microbial communities makes this approach directly relevant to sustainable crop production. Microbes occupy a pivotal role in soil health by their multitrophic functions in nutrient cycling, plant resilience, climate change mitigation, and even pandemic preparedness.

### 6.1. Key Insights

1. Soil-Plant Health and Fertility through Microbial Partnerships: Microbes are fundamental to maintaining soil structure, nutrient availability, and overall soil health, forming the foundation of sustainable agriculture. Plants depend on these microbial functions to optimise root growth, nutrient uptake, and long-term productivity, forming the foundation of sustainable agriculture.
2. Ecosystem Sustainability through Microbial Functions: Microbial communities contribute significantly to carbon sequestration, methane reduction, and ecosystem resilience, positioning them as key players in climate change mitigation strategies. In plants, these processes are enhanced through root exudates and canopy–soil feedback that fuel microbial activity.
3. Plant Health, Productivity and Food Security through Microbial Innovations: The microbial 3Bs—biofertilisers, biostimulants, and bioprotectants—offer sustainable alternatives to chemical inputs, enhancing crop yields while reducing environmental impact. When tailored to crop species and integrated with plant growth stages, these tools deliver maximum yield and quality benefits.
4. Advancing Standardisation and Stewardship in Microbial Agriculture: Emerging technologies in metagenomics, synthetic biology, and precision agriculture are unlocking new potential in microbial applications. These advances will allow real-time optimisation of plant–microbe interactions in field conditions.

### 6.2. Challenges and Opportunities

While the potential of microbiome-based approaches is vast, several challenges remain:

1. Standardisation: Developing globally accepted standards for microbial product quality and efficacy.
2. Scalability: Translating laboratory successes to field-scale applications, especially ensuring that field conditions, plant varieties, and local agronomy align with microbial requirements.
3. Knowledge Gaps: Understanding complex microbial interactions in diverse ecosystems with a specific focus on how these interactions vary among plant species and genotypes.
4. Regulatory Frameworks: Establishing adaptive policies that balance innovation with safety, including safeguards to prevent disruption of beneficial plant–microbe symbioses.

**Figure 4:**
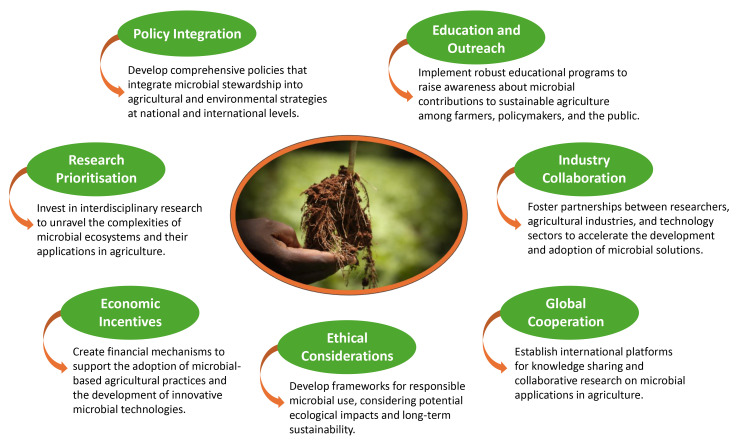
Proposed action points to fully harness the transformative potential of microbiomes in agriculture.

We have proposed a few action points to fully harness the transformative potential of microbiomes in agriculture (Figure 4). By embracing this microbiome-centric approach, we stand at the threshold of a new agricultural era—one that promises not only to meet the food security challenges of the 21st century but also to restore and enhance the health of our planet’s ecosystems. The journey ahead requires collective effort, innovative thinking, and unwavering commitment to sustainable practices. As we move forward, let us view microbes not merely as tools, but as partners with plants as their co-creators and beneficiaries—in our quest for a resilient, productive, and environmentally harmonious agricultural future.

## Figures and Tables

**Table 1:** Summary of microbe–crop–practice linkages and reported effect sizes in climate change mitigation.

Microorganism	Crop System	Agricultural Practice	Reported Effect Size	Key Outcomes	References
Type I & II Methanotrophs	Rice	Alternate wetting & drying (AWD)	↓ CH_4_ emissions by 30–70%; methanotrophic activity significantly ↑ during dry phases	Reduced GHG emissions	[80]
Methanotrophic Bioinoculants	Rice, wetland crops	Inoculation with methanotroph consortia	CH_4_ ↓ 20–40%; ↑ plant growth (PGPR traits)	Grain yield ↑ up to 38%	[81]
Methanotrophic bacteria	Rice	Inoculation (MT-22) with water-management optimisation	↓ CH_4_ ~10–12% (field trials)	Reduced GHG emissions	[82]
Blue-green algae + *Azolla*	Rice	Blue-green algae @ 10 kg ha^−1^ (T_2_), *Azolla* @ 1 tonnes per hectare	CH_4_ emission ↓ 37.9% over the control	Reduced GHGemissions	[83]
Cyanobacteria (biocrust/soil)	Dryland soils	Application of cyanobacteria as inoculants	↑ Soil C sequestration; ↑ aggregate stability	Dryland restoration	[84]
Arbuscular mycorrhizal fungi (AMF)	Wheat, maize	Reduced tillage, organic amendments	↑ SOC stabilization	Aggregate formation ↑ by 15–30%	[85]
Microbial consortia (bioinoculants)	Rice, maize	Inoculation with carbon-sequestering strains	Potential SOC ↑ 12–25%	Carbon sequestration and climate mitigation	[86]
Actinomycetes & Saprophytic fungi	Diverse cropping systems	Compost addition, residue retention	↑ Carbon use efficiency (CUE) and microbial necromass, SOC ↑ by 8–15%	Improved soil health	[87]

↑ = Increase; ↓ = Decrease

## Data Availability

No new data were created or analyzed in this study. Data sharing is not applicable.

## Abbreviations

AMF	Arbuscular mycorrhizal fungi
3Bs	Biofertilisers, biostimulants, and biopro-tectants
CUE	Carbon use efficiency
GHG	Greenhouse gases
ISR	Induced systemic resistance
MICP	Microbe-induced carbonate precipitation
PAHs	Polycyclic aromatic hydrocarbons
PGPR	Plant growth-promoting rhizobacteria
SOC	Soil organic carbon
